# Investigating Sex Differences in Opioid Use Disorder Risk Factors: Insights from Cross-Section Lebanese Study Population

**DOI:** 10.1192/j.eurpsy.2024.273

**Published:** 2024-08-27

**Authors:** K. Chamoun, P. Salameh, H. Sacre, J. Mouawad, L. Rabbaa, B. Megarbane, A. Hajj

**Affiliations:** ^1^Laboratoire de Pharmacologie, Pharmacie Clinique et Contrôle de Qualité des Médicaments; ^2^Faculty of Pharmacy, Saint Joseph University, Beirut, Lebanon; ^3^Inserm, UMR-S1144, Université Paris Cité, Paris, France; ^4^INSPECT-LB (Institut National de Santé Publique, d’Épidémiologie Clinique et de Toxicologie-Liban), Beirut; ^5^School of Medicine, Lebanese American University, Byblos, Lebanon; ^6^Department of Primary Care and Population Health, University of Nicosia Medical School, Nicosia, Cyprus; ^7^Faculty of Pharmacy, Lebanese University, Hadat, Lebanon; ^8^Faculty of Pharmacy, Université Laval; ^9^Oncology Division, CHU de Québec- Université Laval Research Center, Quebec, Canada

## Abstract

**Introduction:**

Opioid use disorder (OUD) is a significant public health concern, and understanding the risk factors associated with OUD is crucial for effective prevention and management strategies. However, limited information is available regarding the role of sex differences in OUD risk factors. Women have often been excluded from clinical studies to create more homogeneous samples and simplify the analysis of treatment effects. The underrepresentation of women in clinical trials and the lack of sex stratification, typically limited to binary comparisons without considering gender dynamics, raise concerns about potential sex disparities. Given the emerging evidence suggesting the possibility of sex differences in the likelihood of developing OUD, further research is needed to investigate and understand these potential disparities to optimize the individualized management of OUD.

**Objectives:**

The primary objective of this study was to examine and identify any sex-related variations in OUD risk variables within the Lebanese community. By pinpointing sociodemographic, psychiatric, and other factors related to sleep and chronotype, we aim to elucidate their impact on the onset and progression of OUD in both males and females.

**Methods:**

A cross-sectional study was conducted among 581 Lebanese adults using an online questionnaire that included sociodemographic questions, validated scales for substance use disorders and sleep disorders, and assessments for depression and anxiety. Multivariate analyses were performed to identify associations between risk factors and OUD scores in both male and female populations.

**Results:**

Common risk factors for OUD were identified, including family and personal history of substance use disorder, co-occurrence of sedative and alcohol misuse, and psychiatric illnesses. Sex-specific risk factors were also observed. Among women, the ASSIST-opioids subscore was significantly associated with the Pittsburgh Sleep Quality Index (B=0.143) and Insomnia Severity Index (B=0.286) scores. Men demonstrated a correlation between ORT-OUD and younger age (B=0.882). Waterpipe consumption was negatively correlated with the ORT-OUD score in men (B=-0.018).

**Conclusions:**

Our study emphasizes the importance of examining sex differences in risk factors for OUD, particularly within the Lebanese population. By acknowledging these gender-specific risk factors, interventions can be customized to address the distinct vulnerabilities of each sex. This approach could potentially improve prevention efforts, facilitate early identification, and implement treatment strategies tailored to the specific needs of individuals with OUD. Further research is needed to delve into the underlying mechanisms and develop targeted interventions for enhanced management of OUD.

**Disclosure of Interest:**

None Declared

